# Choline-stabilized orthosilicic acid supplementation as an adjunct to Calcium/Vitamin D3 stimulates markers of bone formation in osteopenic females: a randomized, placebo-controlled trial

**DOI:** 10.1186/1471-2474-9-85

**Published:** 2008-06-11

**Authors:** Tim D Spector, Mario R Calomme, Simon H Anderson, Gail Clement, Liisa Bevan, Nathalie Demeester, Rami Swaminathan, Ravin Jugdaohsingh, Dirk A Vanden Berghe, Jonathan J Powell

**Affiliations:** 1 Twin Research and Genetic Epidemiology Unit, St Thomas' Hospital, Kings College, London, UK; 2 Department of Pharmaceutical Sciences, Faculty of Pharmaceutical, Biomedical and Veterinary Sciences, University of Antwerp, Antwerp, Belgium; 3 Department of Gastroenterology, College House, South Wing, St Thomas' Hospital, London, UK; 4 Department of Chemical Pathology, North Wing, St Thomas' Hospital, London, UK; 5 Gastrointestinal Laboratory, Rayne Institute (King's College London), St Thomas' Hospital, London, UK; 6 Department of Nutrition and Dietetics, King's College London, 150 Stamford Street, London, UK; 7 MRC Human Nutrition Research, Elsie Widdowson Laboratory, Cambridge, UK

## Abstract

**Background:**

Mounting evidence supports a physiological role for silicon (Si) as orthosilicic acid (OSA, Si(OH)_4_) in bone formation. The effect of oral choline-stabilized orthosilicic acid (ch-OSA) on markers of bone turnover and bone mineral density (BMD) was investigated in a double-blind placebo-controlled trial.

**Methods:**

Over 12-months, 136 women out of 184 randomized (T-score spine < -1.5) completed the study and received, daily, 1000 mg Ca and 20 μg cholecalciferol (Vit D3) and three different ch-OSA doses (3, 6 and 12 mg Si) or placebo. Bone formation markers in serum and urinary resorption markers were measured at baseline, and after 6 and 12 months. Femoral and lumbar BMD were measured at baseline and after 12 months by DEXA.

**Results:**

Overall, there was a trend for ch-OSA to confer some additional benefit to Ca and Vit D3 treatment, especially for markers of bone formation, but only the marker for type I collagen formation (PINP) was significant at 12 months for the 6 and 12 mg Si dose (*vs*. placebo) without a clear dose response effect. A trend for a dose-corresponding increase was observed in the bone resorption marker, collagen type I C-terminal telopeptide (CTX-I).

Lumbar spine BMD did not change significantly. Post-hoc subgroup analysis (baseline T-score femur < -1) however was significant for the 6 mg dose at the femoral neck (T-test). There were no ch-OSA related adverse events observed and biochemical safety parameters remained within the normal range.

**Conclusion:**

Combined therapy of ch-OSA and Ca/Vit D3 had a potential beneficial effect on bone collagen compared to Ca/Vit D3 alone which suggests that this treatment is of potential use in osteoporosis. NTR 1029

## Background

Osteoporosis has become a leading cause of morbidity and mortality worldwide and is an ever-increasing drain on healthcare resources. Osteoporosis is defined as progressive skeletal disorder, characterised by low bone mass (osteopenia) and micro-architectural deterioration, resulting in an increase in bone fragility and increased risk to factures [1]. The aetiology of osteoporosis is multifactorial [2,3] with influences from genetics, endocrine function, exercise and nutrition [4]. The primary cause for the decline in bone mineral density (BMD) and increased susceptibility to fracture in women, is the decrease in circulating estrogens at the onset of menopause.

With hormone replacement therapy's diminishing popularity [5,6] there is a clinical need for alternative well-tolerated pharmacological or nutritional treatments that can safely be used soon after the menopause and which effectively prevent bone loss and the development of osteoporosis [7]. In this regard several studies have looked at the role of bone minerals (magnesium [8] and fluoride [9,10]) and nutritional trace elements (zinc [11,12], copper [11], manganese [13]) including silicon, in bone homeostasis. Experimental silicon deprivation in rats [14-16] and chicks [17,18] demonstrated marked effects on growth and bone metabolism which in some studies resulted in aberrant connective tissue and bone mineralization (thinner cortex, less calcified bone matrix) and bone defects. Keeting [19] and Schütze [20] reported that the silicon-containing compound zeolite (sodium zeolite A) stimulates DNA synthesis in osteoblasts and inhibits osteoclast-mediated bone resorption *in vitro*. Furthermore, in 1992 Mourkarzel [21] reported that a decreased serum Si concentration in total parenterally fed infants was associated with a decreased bone mineral content compared to healthy controls. This was the first observation of a possible dietary silicon deficiency in humans. Silicon supplementation, on the other hand, was shown to have beneficial effects on bone. In a small intervention study [22] treatment of osteoporotic subjects with silicon in the form of monomethyltrisilanol increased trabecular bone volume compared to non-treated controls. In another open intervention study [23] femoral density was significantly increased after intramuscular administration of silicon (50 mg), again, as monometyltrisilanol twice a week for 4 months. An epidemiological study reported a positive correlation between dietary Si intake and bone mineral density at the hip in men and pre-menopausal women, suggesting that a higher Si intake may have a beneficial effect on cortical bone health [24]. More recently the same research group [25] demonstrated that dietary silicon intake is positively associated with BMD in postmenopausal women taking hormone replacement therapy (HRT), suggesting a possible interaction between estrogen status and effects of silicon on bone.

Orthosilicic acid (OSA), also known as soluble silica, is present in low concentrations (< 10^-3 ^M) in beverages and water. Dietary silicates undergo hydrolysis, forming OSA that is readily absorbed in the gastrointestinal tract [26]. Physiological concentrations of OSA were recently found to stimulate collagen type I synthesis and osteoblastic differentiation in human osteoblast-like cells in vitro [27]. Supplementation of young animals with a specific choline-stabilized orthosilicic acid complex (ch-OSA), a concentrated and stabilized source of OSA, resulted in a higher collagen concentration in the skin [28] and an increased femoral bone density [29]. Treatment of humans with oral ch-OSA for 20 weeks, resulted in a significant positive effect on skin surface and skin mechanical properties [30], suggesting a regeneration or *de novo *synthesis of collagen fibers. Furthermore, Calomme *et al*. investigated the effect of ch-OSA supplementation (30 weeks) on bone loss in aged ovariectomized (OVX) rats [31]. The increase in bone turnover in OVX rats tended to be reduced by ch-OSA supplementation. BMD was significantly increased at two sites in the distal femur in OVX rats supplemented with ch-OSA compared to OVX controls. This study demonstrated that ch-OSA supplementation partially prevents femoral bone loss in the aged OVX rat model.

Considering the suggested role of silicon in bone mineralization, ch-OSA may be useful as a preventive or therapeutic agent against osteoporosis in combination with calcium and vitamin D. In the present trial we evaluated the effect of oral choline-stabilized orthosilicic acid on markers of bone turnover and on bone mineral density in osteopenic women.

## Methods

### Subjects

184 osteopenic, but otherwise healthy, Caucasian women with a T-score < -1.5 at the lumbar spine by DEXA scan were recruited by local advertising in St Thomas' Hospital, London. Screening occurred over approximately 24 months and the study was conducted between June 2001 and February 2004. The study protocol was approved by St. Thomas' Hospital Local Research Ethics Committee and all the women gave written informed consent prior to commencing the study. Patients were excluded according to the following criteria: renal failure as defined by serum creatinine > 200 μmol/L, abnormal serum ferritin level (normal range: 11–250 μg/L), concomitant medication (treatment with phosphate-binding antacids > 6 months/year), oral glucocorticoid treatment (> 8 months in the previous year and > 7.5 mg/day prednisone equivalent, or a total dose of more than 2 g prednisone equivalent in the previous 12 months), local injectable glucocorticoid treatment if > 5 injections per year, inhaled glucocorticoid treatment if > 6 months in the previous year and more than 2 mg/day prednisone equivalent (glucocorticoids by local topical administration were not excluded), concomitant or previous treatment for bone diseases (fluoride salts: > 10 mg/day, for more than 2 weeks in the previous 12 months, biphosphanates: for more than 2 weeks in the previous 12 months, oral estrogens, estradiol vaginal ring, anti-estrogens, progesterones, anabolic steroids in the previous 3 months or used for more than 1 month in the previous 6 months, estradiol implants in the previous 3 years, ipriflavone use in the previous 6 months or used for more than 1 month in the previous 12 months, calcitonin use in the previous month or used for more than 1 month in the previous 6 months, other drugs for bone disease currently in development), concomitant and previous use of food supplements containing silicon or horsetail herb extract, bamboo extract, colloidal silicic acid, or silanol derivatives in the previous 6 months.

All study methods and procedures were conducted in accordance with the ethical standards of the Declaration of Helsinki and Good Clinical Practice guidelines.

### Study medication

Subjects who meet the inclusion and exclusion criteria were randomly assigned to four groups to take by oral route ch-OSA (Bio Minerals N.V., Belgium) or a placebo (a choline-glycerol solution without ch-OSA but with identical pH, colour and taste to ch-OSA; Bio Minerals N.V., Belgium) daily for 12 months. Randomization of patient number was performed by Bio Minerals n.v. with GraphPad Software (GraphPad Software Inc., San Diego, USA). Patients received a randomization number e.g. from 001 to 184 in ascending order. Three different ch-OSA doses (3, 6 and 12 drops) were used corresponding to 3, 6, and 12 mg Si, which would increase dietary Si intakes by 16.4, 33, and 66% in this age and gender group [32]. The placebo group was divided in 3 subgroups (3, 6, and 12 drops) to mimic the three different ch-OSA dosages.

The study medication was delivered in sealed 30 ml plastic bottles. The subjects were instructed to mix the ch-OSA or placebo drops with 50 ml (2 floz, 1/4 glass) water or juice and to consume immediately, preferably 30 minutes before a meal or 2 hours after a meal. All subjects received calcium and vitamin D3 (Calcichew/D3 forte, Shire, UK) containing 1000 mg calcium and 20 μg cholecalciferol daily.

The subjects returned their medication at each visit and received new medication for a next study period of 3 months. Patient compliance was assessed at each visit by quantifying the amount of study medication returned. Patients and investigative site staff were blinded to group assignment throughout the study (i.e. double-blinded).

### Measurements

A basic clinical examination was performed at each visit including measurement of body weight, height, systolic and diastolic blood pressure and heart rate. Blood samples and single void urine samples were collected from fasting subjects at baseline and after 12 months supplementation to evaluate the safety parameters such as serum glucose, urea, creatinine, uric acid, ferritin, total protein, cholesterol, triglycerides, HDL-cholesterol, LDL-cholesterol and total bilirubin, glutamic-oxalacetic transaminase (GOT), glutamic-pyruvic transaminase (GPT), gamma-glutamyltransferase (gamma-GT), cholinesterase, trypsin, amylase, and lipase. Other serum parameters analysed were sodium, potassium, calcium, phosphorus, copper, magnesium, 25-OH-vit D3 and zinc. The following parameters were analysed in urine: glucose, proteins, ketones, bilirubin, urobilinogen blood, nitrite, leucocyte esterase, pH, urea, uric acid, creatinine, sodium, potassium, calcium, phosphorus and magnesium. All serum and urine parameters were measured at baseline and after 12 months supplementation.

Bone mineral density (BMD) was assessed by Dual-Energy X-ray Absorptiometry (DEXA) using a Hologic QDR 4500 W (Waltham, MA). Scans of the lumbar spine (L1 to L4) and femur (neck, trochanter, intertrochanteric area, Ward's triangle and total) were performed at screening and/or at the inclusion visit and then after 12 months treatment at the final visit. All measurements for identical subjects were made on the same densitometer throughout the study.

Biochemical markers of bone formation (osteocalcin (OC), bone specific alkaline phosphatase (BAP), procollagen type I N-terminal propeptide (PINP)) and bone resorption (deoxypyridoline (DPD), C-terminal telopeptide of type I collagen (CTX-I)) were measured at baseline and after 6 and 12 months of treatment. PINP was measured by competitive radio-immunoassay (Orion Diagnostic PINP RIA kit) and osteocalcin by competitive ELISA (Metra Osteocalcin EIA). BAP and DPD were measured by a competitive immunoassay (Metra BAP EIA and Metra DPD EIA, respectively). Urine CTX-I was measured using the Nordic Bioscience Diagnostics™ serum CrossLaps^® ^ELISA assay kit (Denmark).

### Statistical analysis

Results are expressed as means ± standard deviation (SD). Outliers, defined as > (median+2SD) or < (median-2SD) were omitted from bone marker analysis. Comparison of multiple means was carried out by multiple covariate analysis, adjusted for baseline values (bone markers, BMD, MANCOVA). Differences between two groups (% change from baseline) were evaluated with a t-test. Post-hoc subgroup analysis was performed on a subpopulation with osteopenia of the hip (T score < -1). All tests are two-sided and p <0.05 was defined as significant. Considering a biological variation for bone markers of 35%, a group size of 35 subjects per group/arm was calculated to observe a significant difference (p < 0.05) in bone markers of 25% between ch-OSA and placebo groups with a power of 85%. A drop out rate of 20% was also taken into account when determining the total number of patients that had to be included (n = 175). Analysis was performed using SPSS software (version 13.0, Chicago, USA).

## Results

The study started with 184 mostly post-menopausal (85%) women, mean age 60.7 ± 10.4 years, with documented osteopenia at the lumbar spine (T-score < -1.5), of which 136 completed the study (37 in the placebo group and 33 in each of the ch-OSA groups, see additional file 1 for more information). Baseline characteristics are represented in Table 1. There were no significant differences between the groups, except that the 12 mg Si group had statistically significant lower lumbar spine BMD (*vs*. 3 mg Si group) and higher urinary DPD level (*vs*. placebo) at baseline. The mean compliance (± SD) in the three ch-OSA dosing groups was respectively 110% (± 40%, 3 mg Si in 3 drops), 110% (± 30%, 6 mg Si in 6 drops), and 101% (± 7%, 12 mg Si in 12 drops). The compliance in the placebo groups was respectively 123% (± 13%, 3 drops), 107% (± 12%, 6 drops) and 100% (± 12%, 12 drops).

**Table 1:** Baseline characteristics

	**Placebo**	**ch-OSA**		
		**3 mg Si**	**6 mg Si**	**12 mg Si**
**AGE (years)**	62.0 ± 10.9 (n = 37)	60.4 ± 11.8 (n = 33)	59.7 ± 9.4 (n = 33)	60.8 ± 9.7 (n = 33)
**BMI**	23.2 ± 3.0 (n = 37)	24.4 ± 3.8 (n = 33)	24.1 ± 4.6 (n = 33)	25.2 ± 3.8 (n = 33)
**Menopausal status**	6 pre/31 post (n = 37)	6 pre/27 post (n = 33)	5 pre/28 post (n = 33)	4 pre/29 post (n = 33)
				
**BMD**				
**Spine total (g/cm^2^)**	0.797 ± 0.061 (n = 37)	0.805 ± 0.052 (n = 33)	0.805 ± 0.069 (n = 33)	0.776 ± 0.072^b ^(n = 33)
**Femur total (g/cm^2^)**	0.789 ± 0.103 (n = 37)	0.791 ± 0.094 (n = 33)	0.792 ± 0.080 (n = 33)	0.781 ± 0.103 (n = 33)
**Femur neck (g/cm^2^)**	0.670 ± 0.085 (n = 37)	0.677 ± 0.085 (n = 33)	0.667 ± 0.079 (n = 33)	0.649 ± 0.084 (n = 33)
				
**BONEMARKERS**				
**OC (ng/ml)**	9.74 ± 3.16 (n = 31)	10.17 ± 3.91 (n = 27)	10.65 ± 3.32 (n = 27)	10.16 ± 3.67 (n = 25)
**BAP (U/L)**	21.65 ± 6.30 (n = 32)	20.43 ± 7.24 (n = 26)	21.85 ± 6.31 (n = 27)	21.58 ± 7.36 (n = 27)
**PINP (μg/L)**	47.20 ± 13.45 (n = 30)	46.28 ± 21.46 (n = 26)	44.78 ± 16.07 (n = 28)	48.77 ± 15.09 (n = 30)
**DPD (/Cr)**	5.57 ± 3.12 (n = 28)	5.22 ± 1.50 (n = 26)	5.74 ± 1.45 (n = 27)	5.92 ± 1.57^a ^(n = 28)
**CTX-I (μg/mmol Cr)**	359.01 ± 129.89 (n = 31)	331.88 ± 155.87 (n = 26)	349.10 ± 126.71 (n = 27)	360.43 ± 139.00 (n = 28)

Baseline characteristics of patients (n = 136), mean ± SD, a: p < 0.05 (unpaired t-test) versus placebo, b: p < 0.05 (unpaired t-test) versus 3 mg Si.

### Safety and tolerability

Biochemical safety parameters were analysed in serum (Table 2) and urine (Table 3) at baseline and after 12 months treatment. Forty-eight subjects did not complete the study and reasons for withdrawal were medical or volunteer decision (non-medical) i.e. volunteer changing their mind as to taking further part in the study. Four cases were classified as serious adverse events: neuro-endocrine tumor combined with liver cancer (6 mg Si group), liver cancer combined with gall bladder disease (6 mg Si group), breast cancer (6 mg Si group), and cerebro-vascular accident (12 mg Si group). In three of these four cases a disturbed liver function was observed at baseline, prior to the start of the study. After considering the specific pathology, these serious adverse events were reported as not related to the study medication. No ch-OSA related adverse events were observed.

**Table 2:** Biochemical serum safety parameters

	**Normal range**		**Placebo (n = 37)**		**ch-OSA**					
					**3 mg Si (n = 33)**		**6 mg Si (n = 33)**		**12 mg Si (n = 33)**	
	**LL**	**UL**	**Baseline**	**T12**	**Baseline**	**T12**	**Baseline**	**T12**	**Baseline**	**T12**
**Serology**										
**Glucose (mg/dL)**	70	110	87,43 ± 8,41	87,27 ± 6,74	89,03 ± 14,39	88,00 ± 10,32	86,50 ± 8,08	84,38 ± 9,23	86,16 ± 10,16	86,69 ± 9,72
**Urea (mg/dL)**		50,1	32,27 ± 8,66	32,39 ± 8,60	30,05 ± 8,51	32,46 ± 7,40	28,07 ± 7,45	30,33 ± 8,17(a)	31,71 ± 7,12	30,54 ± 5,93
**Creatinine (mg/dL)**	0,60	1,40	0,79 ± 0,13	0,83 ± 0,11(a)	0,76 ± 0,14	0,84 ± 0,13(a)	0,72 ± 0,12	0,79 ± 0,11(a)	0,80 ± 0,12	0,82 ± 0,09
**Uric acid (mg/dL)**	2,6	7,2	5,53 ± 1,07	5,52 ± 1,00	5,92 ± 1,38	6,23 ± 1,51	5,25 ± 1,07	5,09 ± 1,05	5,88 ± 1,01	5,71 ± 0,99
**Ferritin (μg/L)**	11	250	60,24 ± 38,75	52,38 ± 33,81(a)	63,84 ± 37,24	59,72 ± 36,12	44,97 ± 26,98	43,97 ± 32,96	77,09 ± 57,98	66,47 ± 49,59(a)
**Total proteins (g/dL)**	6,4	8,3	7,14 ± 0,51	7,08 ± 0,37	7,00 ± 0,67	7,15 ± 0,32	7,20 ± 0,44	7,00 ± 0,32(a)	7,21 ± 0,36	7,13 ± 0,49
**Cholesterol (mg/dL)**		190	241,59 ± 52,41	236,03 ± 52,09	223,22 ± 49,28	226,53 ± 34,47	241,47 ± 35,61	224,69 ± 26,44 (a)	238,25 ± 35,48	226,69 ± 33,52
**Triglycerides (mg/dL)**		180	105,49 ± 41,12	107,89 ± 51,95	130,28 ± 140,94	108,63 ± 61,67	100,59 ± 41,99	100,38 ± 40,21	101,81 ± 52,76	116,19 ± 110,20
**HDL-cholesterol (mg/dL)**	40		50,11 ± 12,59	54,86 ± 12,58 (a)	48,84 ± 18,54	55,28 ± 15,43 (a)	53,13 ± 15,57	54,59 ± 12,94	47,53 ± 14,14	52,69 ± 14,58(a)
**LDL-cholesterol (mg/dL)**		115	169,95 ± 47,83	159,11 ± 46,14 (a)	152,87 ± 45,41	150,90 ± 35,71	167,88 ± 31,09	149,69 ± 24,62 (a)	169,61 ± 31,09	152,74 ± 34,18(a)
**HDL/LDL**			0,32 ± 0,12	0,38 ± 0,15(a)	0,36 ± 0,17	0,40 ± 0,18 (a)	0,33 ± 0,12	0,38 ± 0,12 (a)	0,30 ± 0,10	0,37 0,13 (a)
**Bilirubin total (mg/dL)**	0,1	1,3	0,43 ± 0,17	0,43 ± 0,17	0,39 ± 0,17	0,40 ± 0,16	0,43 ± 0,18	0,38 ± 0,11 (a)	0,46 ± 0,23	0,51 ± 0,33
**SGOT (AST) (U/L)**		37	11,27 ± 3,44	13,11 ± 3,03(a)	11,59 ± 4,15	13,44 ± 4,41(a)	10,88 ± 2,88	12,34 ± 3,46 (a)	11,28 ± 3,99	12,72 ± 3,74(a)
**SGPT (ALT) (U/L)**		38	9,57 ± 4,38	8,00 ± 4,01(a)	9,91 ± 5,29	8,69 ± 4,50	8,75 ± 4,44	8,09 ± 2,91	10,16 ± 5,12	8,56 ± 3,75 (a)
**SGOT/SGPT**			1,38 ± 0,68	1,99 ± 0,91(a)	1,46 ± 0,75	1,93 ± 0,97(a)	1,55 ± 0,78	1,73 ± 0,78	1,46 ± 0,51	1,71 ± 0,74(a)
**GGT (U/L)**		57	27,89 ± 19,64	25,27 ± 12,71	31,13 ± 41,16	31,19 ± 46,32	30,75 ± 56,18	34,31 ± 71,67	34,72 ± 44,76	34,88 ± 42,45
**Cholinesterase (U/L)**	3930	11500	7785,5 ± 1547,5	7517,7 ± 1582,5	7235,4 ± 1987,2	7425,44 ± 1628,08	7356,2 ± 1668,9	7061,4 ± 1366,8	7178,8 ± 1320,6	7063,3 ± 1075,9
**Amylase (U/L)**		100	57,03 ± 17,15	56,22 ± 15,92	115,81 ± 317,01	128,16 ± 372,96	59,13 ± 22,08	58,53 ± 22,46	63,88 ± 21,48	62,66 ± 21,72
**Lipase (U/L)**	7	60	30,22 ± 15,18	27,35 ± 8,30	32,25 ± 14,01	32,09 ± 12,64	31,00 ± 15,38	29,44 ± 14,72	30,59 ± 13,29	28,19 ± 10,31
**Trypsin (μg/L)**	10	57	44,23 ± 12,81	46,31 ± 10,56	49,19 ± 12,50	52,21 ± 12,78	44,58 ± 10,36	48,49 ± 18,86	43,86 ± 12,36	45,14 ± 11,74
**Sodium (mmol/L)**	135	145	139,70 ± 3,65	139,89 ± 5,29	137,03 ± 6,99	140,63 ± 1,90 (a)	139,75 ± 4,18	140,59 ± 2,56	140,00 ± 2,87	140,63 ± 2,57
**Potassium (mmol/L)**	3,5	5,1	3,99 ± 0,24	3,97 ± 0,25	3,89 ± 0,36	3,96 ± 0,24	3,86 ± 0,25	3,98 ± 0,21 (a)	3,90 ± 0,22	3,89 ± 0,25
**Calcium (mg/L)**	86	100	91,81 ± 5,35	92,73 ± 5,89	90,69 ± 9,42	93,69 ± 6,40	93,16 ± 4,01	93,31 ± 4,59	94,50 ± 3,32	93,41 ± 4,32
**Phosphorus (mg/dL)**	2,7	4,5	3,60 ± 0,44	3,70 ± 0,43	3,53 ± 0,55	3,65 ± 0,46	3,59 ± 0,43	3,70 ± 0,43	3,62 ± 0,40	3,63 ± 0,44
**Cupper (μg/dL)**	70	155	91,57 ± 14,43	108,19 ± 16,23 (a)	101,50 ± 35,66	111,44 ± 24,69 (a)	85, 48 ± 13,75	101,81 ± 16,70 (a)	93,56 ± 15,15	110,63 ± 14,03 (a)
**Magnesium (mg/dL)**	1,6	2,5	2,06 ± 0,14	1,95 ± 0,17(a)	1,98 ± 0,22	1,92 ± 0,17	2,05 ± 0,20	1,92 ± 0,14 (a)	1,99 ± 0,18	1,92 ± 0,18 (a)
**Zinc (μg/dL)**	50	120	67,59 ± 7,77	75,08 ± 11,00 (a)	66,19 ± 10,64	72,63 ± 11,96 (a)	64,50 ± 9,80	68,63 ± 8,17 (a)	63,00 ± 7,34	67,00 ± 9,42
**25-OH-vit. D3 (ng/mL)**	6,3	46,4	19,26 ± 7,30	25,43 ± 8,03 (a)	17,02 ± 7,21	26,31 ± 5,47(a)	16,76 ± 9,62	25,83 ± 8,40 (a)	18,78 ± 9,63	26,21 ± 6,52 (a)

Baseline biochemical safety parameters in serum and after 12 months treatment with placebo (Ca/vit D) and three different ch-OSA doses (Si + Ca/vit D; Si: 3, 6, 12 mg/day), mean ± SD. LL: Lower limit; UL: Upper limit; (a) p < 0.05 versus baseline (two-tailed t-test).

**Table 3:** Biochemical urinary safety parameters

	**Normal range**		**Placebo (n = 37)**		**ch-OSA**					
					**3 mg Si (n = 33)**		**6 mg Si (n = 33)**		**12 mg Si (n = 33)**	
	**LL**	**UL**	**Baseline**	**T12**	**Baseline**	**T12**	**Baseline**	**T12**	**Baseline**	**T12**
Urine analysis*										
Glucose*			0	0	0	1	0	0	0	0
Proteins*			2	0	0	0	1	0	1	2
Ketons*			0	0	0	0	0	0	2	0
Bilirubine*			0	0	0	0	0	0	0	0
Urobilinogene*			0	0	0	0	0	0	0	0
Blood*			1	2	0	0	0	0	0	2
Nitrite*			0	0	0	1	1	0	1	0
Leucocyte esterase*			16	19	9	12	8	6	13	18
***pH***	4,6	8	6,49 ± 0,90	6,28 ± 0,71	6,08 ± 0,89	5,83 ± 0,51	6,20 ± 0,76	6,36 ± 0,84	6,00 ± 0,65	5,98 ± 0,66
***Urea/creatinine***	13,5	32	24,15 ± 7,16	26,41 ± 7,81	24,03 ± 8,22	24,24 ± 9,48	24,69 ± 6,01	24,76 ± 8,32	23,58 ± 7,08	22,13 ± 7,34
***Creatinine (g/L)***	0,60	1,80	0,63 ± 0,36	0,63 ± 0,41	0,63 ± 0,39	0,77 ± 0,45 (a)	0,50 ± 0,29	0,63 ± 0,42	0,66 ± 0,62	0,76 ± 0,63
***Uric acid/creatinine***	0,23	0,68	0,49 ± 0,20	0,47 ± 0,20	0,45 ± 0,19	0,39 ± 0,21	0,50 ± 0,21	0,43 ± 0,18	0,48 ± 0,25	0,42 ± 0,21
***Sodium/creatinine (mmol/g)***	90	200	166,09 ± 68,78	168,15 ± 99,21	178,37 ± 109,89	165,28 ± 157,62	171,38 ± 111,23	162,55 ± 100,52	144,02 ± 85,53	118,99 ± 85,45
***Potassium/creatinine (mmol/g)***	22,7	113,6	78,17 ± 40,39	66,96 ± 33,02	67,20 ± 43,67	54,49 ± 27,96	73,13 ± 46,85	66,05 ± 33,25	66,44 ± 28,54	57,37 ± 27,89
***Calcium/creatinine (mg/g)***	45	273	169,18 ± 85,86	239,76 ± 130,58 (a)	195,61 ± 114,65	268,46 ± 160,38 (a)	240,19 ± 136,88	261,81 ± 135,76	198,32 ± 101,83	190,55 ± 113,90
***Phosphorus/creatinine***	0,36	1,18	0,96 ± 0,31	0,90 ± 0,29	0,96 ± 0,32	0,82 ± 0,31(a)	0,93 ± 0,29	0,81 ± 0,36	0,99 ± 0,26	0,77 ± 0,26 (a)
**Magnesium/creatinine (mg/g)**	64	109	117,32 ± 51,29	125,79 ± 44,99	118,73 ± 57,22	119,54 ± 50,40	131,46 ± 46,21	125,54 ± 54,17	132,52 ± 65,26	110,76 ± 69,66

Baseline urinary biochemical safety parameters and after 12 months treatment with placebo (Ca/vit D) and three different ch-OSA doses (Si + Ca/vit D; Si: 3, 6, 12 mg/day), mean ± SD. LL: Lower limit; UL: Upper limit; * number of patients with parameter present in urine; (a) p < 0.05 versus baseline (two-tailed t-test).

Daily vitamin D3 supplementation significantly increased (within the normal range) serum 25-OH-vit D_3 _in all groups. Baseline values of total serum cholesterol and LDL-cholesterol were higher than the upper limit of the normal range in both the placebo and the three ch-OSA dosing groups. The mean serum amylase concentration was outside the normal range in the 3 mg Si group, both at baseline and after 12 months treatment. Remaining serum parameters were found within the normal range at baseline and after 12 months treatment in all four groups.

Mean baseline urinary magnesium/creatinine ratio was increased (outside normal range) in all four groups (Table 3). Baseline values of other parameters were within the normal range in all groups and continued to be so after 12 months treatment.

Overall some significant differences between start and end of treatment were noted in biochemical markers, but these did not seem related to ch-OSA supplementation (Tables 2 and 3) as, mainly, they occurred across all groups including placebo, suggesting either Ca/vit D related changes or choline-related changes.

### Bone markers

**Figure 1:**
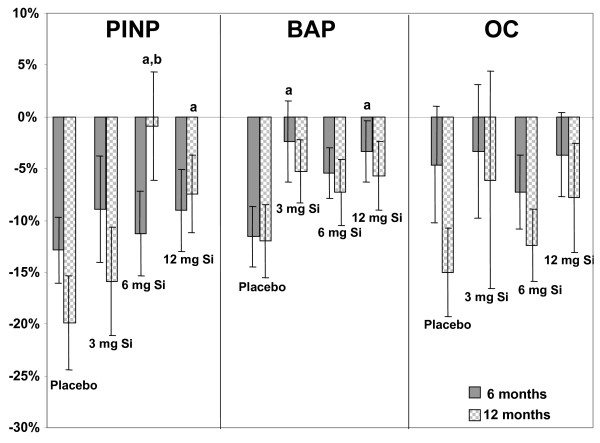
**Change in bone formation markers**. Relative change (mean ± SE, %) in bone formation markers PINP, BAP and OC compared to baseline after 6 and 12 months supplementation with placebo (Ca/vit D) and three different ch-OSA doses (Si + Ca/vit D; Si: 3, 6, 12 mg/day). a: p < 0.05 (t-test) versus placebo; b: p < 0.05 (t-test) versus 3 mg Si.

Baseline bone markers levels were not significantly different between the groups (Table 1), with the exception of DPD, which was higher in the 12 mg Si group compared to the placebo group (p < 0.05, t-test). In all groups, there was a wide variation in bone marker levels after 6 and 12 months (Table 4). However, the variation or change in levels (at 6 and 12 months) compared to baseline were generally less with ch-OSA supplementation compared to placebo (Table 4 & Figure 1); with a trend for a dose-response in many cases (i.e., smallest change at the higher dose). PINP levels were significantly higher after 12 months ch-OSA supplementation (6 and 12 mg Si/day) compared to the placebo group (p < 0.05, MANCOVA; Table 4). Furthermore, the change in PINP levels compared to baseline levels were significantly smaller after 12 months ch-OSA supplementation (6 and 12 mg Si/day) compared to the placebo group (p < 0.05, t-test; Figure 1). A similar trend was observed for BAP reaching statistical significance after 6 months ch-OSA supplementation (p < 0.05, t-test; Figure 1) for 3 and 12 mg Si/day.

**Table 4:** Bone formation and resorption markers

	**Placebo**			**ch-OSA**								
				
				**3 mg Si**			**6 mg Si**			**12 mg Si**		
	*Baseline*	*6 months*	*12 months*	*Baseline*	*6 months*	*12 months*	*Baseline*	*6 months*	*12 months*	*Baseline*	*6 months*	*12 months*

**serum OC (ng/mL)**	9.74 ± 3.16	9.11 ± 3.73^a^	7.86 ± 2.26^a^	10.17 ± 3.91	9.11 ± 3.02^a^	8.33 ± 2.63^a^	10.65 ± 3.32	9.74 ± 3.05^a^	9.08 ± 2.56^a^	10.16 ± 3.67	9.43 ± 3.02^a^	8.79 ± 2.82^a^
**serum BAP (U/L)**	21.65 ± 6.30	18.91 ± 5.56	18.61 ± 5.56	20.43 ± 7.24	19.61 ± 6.66	18.97 ± 5.98	21.85 ± 6.31	20.59 ± 6.39	20.16 ± 6.01	21.58 ± 7.36	20.19 ± 5.62	19.75 ± 5.96
**serum PINP (μg/L)**	47.20 ± 13.45	40.83 ± 14.05^a^	36.49 ± 12.04^a^	46.28 ± 21.46	40.27 ± 20.18^a^	35.63 ± 12.34^a^	44.78 ± 16.07	38.60 ± 13.35^a^	42.04 ± 12.77^a,1,2^	48.77 ± 15.09	42.87 ± 11.89^a,2^	43.73 ± 12.11^a,1,2^
**serum DPD (/Cr)**	5.57 ± 3.12	4.77 ± 1.12	4.74 ± 1.28	5.22 ± 1.50	5.17 ± 2.04	4.83 ± 1.62	5.74 ± 1.45	5.81 ± 1.68	5.49 ± 1.70	5.92 ± 1.57	5.36 ± 1.91	6.22 ± 2.62
**serum CTX-I (μg/mmol Cr)**	359.01 ± 129.89	271.15 ± 124.21	284.66 ± 134.09	331.88 ± 155.87	245.03 ± 107.82	220.71 ± 98.08	349.10 ± 126.71	294.21 ± 137.80^2^	319.38 ± 160.78^2^	360.43 ± 139.00	310.95 ± 122.05^2^	320.99 ± 113.63^2^

Bone formation and resorption markers at baseline (mean ± SD) and after 6 and 12 months supplementation with placebo (Ca/vit D) and three different ch-OSA doses (Si + Ca/vit D; Si: 3, 6, 12 mg/day). a: p < 0.05 (MANCOVA, *vs*. baseline), 1: p < 0.05 (MANCOVA, *vs*. placebo) and 2: p < 0.05 (MANCOVA, *vs*. 3 mg Si).

OC: osteocalcin, BAP: bone specific alkaline phosphatase, PINP: procollagen type I N-terminal propeptide, DPD: deoxypyridinoline, CTX-I: C-terminal collagen type I.

### Bone Mineral Density

Baseline BMD were comparable for all groups, however, baseline BMD at the lumbar spine was significantly lower in the 12 mg Si group compared to the low dose Si group (3 mg Si/day) (p < 0.05, t-test, Table 1). BMD at the femur and lumbar spine did not change significantly after 12 months ch-OSA administration (data not shown). Post-hoc subgroup analysis of femoral neck BMD (baseline femur T-score < -1) was significant at the 6 mg dose compared to placebo which showed a negative change in BMD (p < 0.05, t-test; Table 5).

**Table 5:** Change in bone mineral density

	**Placebo (n = 20)**	**ch-OSA**		
		
		**3 mg Si (n = 18)**	**6 mg Si (n = 19)**	**12 mg Si (n = 24)**
**Spine total**	0.58 ± 2	0.08 ± 3	0.24 ± 2	0.11 ± 3
**Femur total**	-0.54 ± 2	0.07 ± 3	0.44 ± 3	0.04 ± 3
**Femur neck**	-1.22 ± 3	-1.58 ± 3	0.78 ± 3^a,b^	-0.84 ± 2^c^

Relative % change in bone mineral density compared to baseline (mean ± SD), after 12 months supplementation with placebo (Ca/vit D) and three different ch-OSA doses (Si + Ca/vit D; Si: 3, 6, 12 mg/day), in a subgroup of patients with documented osteopenia at the femur. a: p < 0.05 versus placebo, b: p < 0.05 (t-test) versus 3 mg Si and c: p < 0.05 versus 6 mg Si (t-test).

## Discussion

Well-tolerated pharmacological treatments that can effectively prevent bone loss and the development of postmenopausal osteoporosis are needed. The efficacy of all osteoporosis drug treatments is dependent on a sufficient intake of calcium and vitamin D [3], as evidenced by the fact that the pivotal studies underpinning the approval of these drugs required adequate calcium and vitamin D intake [33].

The primary aim of the present study was to investigate whether the combined treatment of ch-OSA with calcium & vitamin D3 (Calcichew/D3 forte) is more efficacious in changing biochemical indices of bone resorption/formation compared to calcium & vitamin D3 alone. Three different ch-OSA doses were investigated as no previous data was available for the optimal/effective ch-OSA dose in man that was bone active. The study was designed to detect differences in bone markers of 25% between the ch-OSA and placebo groups. However, the dropout rate was higher than expected (26%), decreasing the power of the study. Considering the smaller expected change in BMD (1–5%, for existing treatments) after only 12 months of treatment, we expected at best the study to show a trend for an effect of ch-OSA on BMD. Indeed, the overall findings of the study suggest a trend for ch-OSA to confer some additional benefit over Ca & Vit D3 treatment, especially on markers of (bone) collagen metabolism. Few studies [34-36] report the influence of Ca/vitamin D3 on bone markers and show a comparable effect to our subjects treated with placebo.

The major effect of ch-OSA supplementation was on PINP, a marker of type I collagen synthesis and an early marker of bone formation. There was a trend for ch-OSA supplementation to increase PINP synthesis at 12 months, however, the difference was only significant for the 6 and 12 mg doses and without a clear dose response effect. There was also a corresponding increase in serum levels of collagen type I C-terminal telopeptide, a marker of type I collagen degradation, with these ch-OSA doses; again, suggesting ch-OSA may affect (bone) collagen metabolism. In an earlier study of ch-OSA supplementation in young animals, an increase in collagen concentration was shown in the skin [28]. Similarly in a more recent study, oral intake of ch-OSA in humans (10 mg Si/day for 20 weeks) resulted in a significant improvement in skin microrelief and mechanical properties of the skin [30]. This improvement was suggested to be the result of a regeneration or *de novo *synthesis of skin collagen. Previously, Refitt et al. [27] had reported that physiological concentrations of OSA stimulates collagen type I synthesis in human osteoblast-like cells and dermal fibroblasts *in vitro *and, to promote osteoblastic differentiation. Furthermore, during the last years, bioactive silica-containing compounds have been demonstrated to induce collagen synthesis and apatite formation [37-39]. When gels of these materials were applied in orthopaedic surgery, improved healing was demonstrated [40-42]. Orthosilicic acid is present in dissolutions of bioactive silica and was suggested to be the active compound responsible for the biological actions of these preparations. Previously, we have found (unpublished results) that Si is present in the form of orthosilicic acid in serum and urine after the intake of ^29^Si-labelled ch-OSA in humans. Taken into account the effect on PINP and CTX-I, we suggest that ch-OSA supplementation is likely to result in stimulation of type I collagen metabolism in bone. Such a specific effect of ch-OSA on bone collagen metabolism without increasing the synthesis of non-collagenous proteins would explain why no clear effect was found on osteocalcin and BAP levels.

Bone strength depends not only on the quantity of bone mineral (BMD) but also on the quality, which is characterized by several factors including collagen content (and quality). Collagen provides elasticity and structure in all connective tissues and several studies have indicated that collagen is important for bone toughness [43-45] whereas the mineral component is mainly involved in providing stiffness. Wang *et al*. [46] demonstrated that the mechanical integrity of collagen fibres deteriorates with ageing in human cortical bones and is associated with a higher fracture risk. When the collagen network becomes weaker with age, it will result in decreased toughness, possibly due to a reduction in natural cross-links or silicon content. It has previously been suggested that Si may be an integral (structural) component of connective tissues as high levels of non-dialysable Si has been reported in connective tissues and their components suggesting strong (covalent) associations [47]. Thus ch-OSA supplementation may improve the mechanical properties of osteopenic bone by increasing collagen content or improving its quality, however, this was not investigated here.

BMD at the lumbar spine did not change significantly with ch-OSA supplementation, but post-hoc subgroup analysis (subjects with baseline T-score at the femur < -1) showed a significant change in femoral neck BMD with the 6 mg dose compared to placebo (Table 5). After 12 months of combined ch-OSA and Ca/vit D treatment, BMD at the total femur and femoral neck were respectively 0.98% and 2% higher, compared to Ca/vit D alone (placebo group, Table 5). Our study was not designed to assess the effect of ch-OSA on fracture risk, but the increase in femoral BMD may lead to a reduction in the hip fracture risk.

It is unclear why the association between Si intake and lumbar spine BMD is weaker compared with the hip sites, which is the opposite to most trials. This lack of association with lumbar spine could be due to a combination of two factors. Firstly, the insensitivity, or error associated in, measuring spine BMD by DEXA and the reduced power of this study to detect small changes in BMD. In addition, the lumbar spine is the site of artifactual calcification such as degenerative spine changes and vascular calcification, and these could mask and thus weaken the association between Si intake and BMD. Previously, Calomme *et al*. reported that ch-OSA supplementation partially, but significantly ameliorated the decrease in femoral density in ovariectomized-aged rats, whereas the effect at the lumbar spine did not reach significance [31].

The dose of ch-OSA used in this study (3–12 mg Si/day) was low compared to typical daily intake of Si in Western populations (20–50 mg; Pennington [48]). Recently McNaughton et al. [32] reported that mean dietary silicon intake is 18.3 mg in post-menopausal women aged 60–64 years. The mean age of our subjects in this study was 60.7 years and 85% were post-menopausal. Therefore, the three different doses in the present study, 3, 6 and 12 mg Si would typically increase daily dietary Si intakes by 16.4, 33 and 66% respectively, although this has not taken into account relative Si bioavailability of the diet. Previous comparison studies have shown that ch-OSA has high bioavailability compared to other silicon compounds/supplements such as colloidal silicic acid and phytolytic silica [49,50]. The major dietary sources of Si are cereal/whole grain based products and some vegetables and fruits, but modern food processing, including refining, reduces the Si content of the foods and thus dietary Si intakes. Jugdaohsingh et al. [24] reported a significant, positive correlation between dietary silicon intake and BMD at all hip sites in men and pre-menopausal women, suggesting that increased silicon intake is associated with increased cortical BMD in these populations.

Of the 184 women randomised into the study, only 136 completed the study. Reasons for withdrawal were unforeseen illness/medical conditions and some volunteers deciding not to take any further part in the study (non-medical). There were no ch-OSA related adverse events observed during this trial and biochemical safety parameters remained within the normal range. Consequently, oral use of ch-OSA, up to 12 mg Si daily for 12 months, can be regarded as safe.

Total cholesterol and LDL-cholesterol were already increased at baseline, which is most likely due to the consumption of a diet high in cholesterol and saturated fats [51]. Baseline urinary values of magnesium/creatinine ratio were higher than the upper limit of the normal range in all four treatment groups possibly due to a lower muscle mass in osteopenic women compared to healthy subjects [52].

Compliance was comparable for the three dosing groups although the variation was considerable higher for the lowest dose (3 mg). Considering, the galenic form (liquid) this was not unexpected as dosing of drops can be inaccurate, especially in case of low volumes. Therefore solid galenic formulations of ch-OSA may be more preferable.

Finally, choline, the stabilizing agent in ch-OSA might act synergistically with orthosilicic acid on bone. In fact, choline is classified by the Food and Nutrition Board as an essential nutrient. Although humans can synthesize choline in small amounts, dietary sources are needed to prevent deficiency [53]. Choline is a precursor of phospholipids, which are essential components of biological membranes, and is involved in cell signaling and lipid transport/metabolism. Choline was previously reported to minimize leg weakness in broiler chicks [54]. Supplemental choline chloride significantly increased the hexosamine content of the epiphyseal cartilage as compared to cartilage from chicks fed the basal diet. Dietary choline deficiency in rats led to a marked reduction in osteogenesis [55]. Microscopic observation revealed that osteogenesis was lower in rats fed a choline-deficient diet, at both 15 and 30 days, and that this decrease did not revert with a control diet. Histomorphometric evaluation showed 37% and 27% reduction in bone tissue density at 5 and 30 days, respectively, and a 30% decrease in bone formation at 30 days, compared to controls. It is therefore possible that the effect on bone of relatively low doses ch-OSA (compared to the dietary Si intake), observed in the present study, could be in part explained by a synergistic action of orthosilicic acid and choline.

## Conclusion

Accumulated evidence over the last 30 years has suggested a role for Si in bone and connective tissue health. The study presented here suggests that the combined treatment of ch-OSA (Si) with Ca/Vit D3 is safe and has a potentially beneficial effect on bone turnover, especially on bone collagen, and possibly also on femoral BMD compared to Ca/Vit D3 alone. Studies in a larger number of subjects are needed to investigate further the effect of ch-OSA on BMD and its impact on fracture incidence.

## Competing interests

Mario Calomme and Nathalie Demeester received a research grant of Bio Minerals n.v.

## Authors' contributions

TDS, MRC, RJ, DAVB, JJP: concept and design of the study. TDS: principal investigator. TDS, SHA, GC, LB: DEXA scans, medical follow-up and examination of patients. RS: analysis of bone markers and biochemical safety parameters. MRC, ND: data analysis and writing first draft of manuscript. TDS, MRC, ND, RJ, JJP, DAVB: data interpretation.

## Pre-publication history

The pre-publication history for this paper can be accessed here:



## Supplementary Material

Additional file 1 Subject Flow Chart. Chart representing the amount of subject screened, included and completed. Click here for file
