# Supplementary figures and images for: Choline-stabilized orthosilicic acid supplementation as an adjunct to Calcium/Vitamin D3 stimulates markers of bone formation in osteopenic females: a randomized, placebo-controlled trial
Source: BMC Musculoskelet Disord. 2008 Jun 11;9:85. doi: 10.1186/1471-2474-9-85 (PMC2442067; doi:10.1186/1471-2474-9-85)

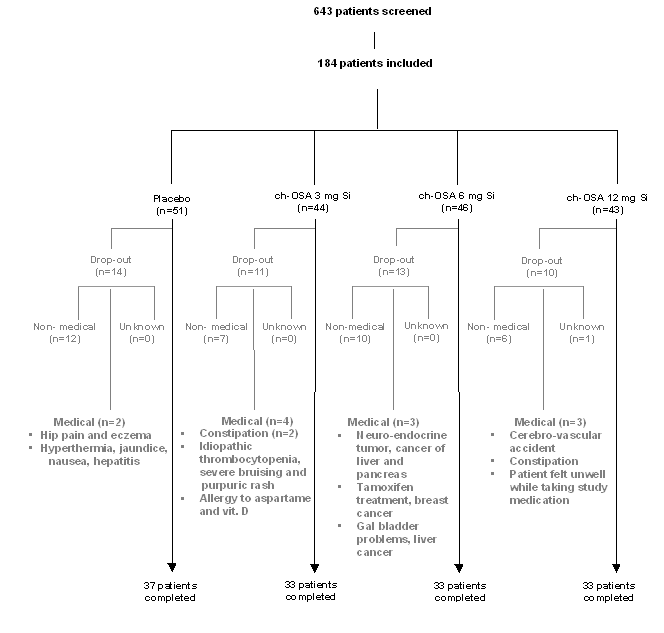
Subject flow chart

Chart representing the amount of subject screened, included and completed.

Supplement: Additional file 1 — Subject Flow Chart. Chart representing the amount of subject screened, included and completed. [file 1471-2474-9-85-S1.doc]
